# Prediction of Neurological Impairment in Cervical Spondylotic Myelopathy using a Combination of Diffusion MRI and Proton MR Spectroscopy

**DOI:** 10.1371/journal.pone.0139451

**Published:** 2015-10-02

**Authors:** Benjamin M. Ellingson, Noriko Salamon, Anthony J. Hardy, Langston T. Holly

**Affiliations:** 1 Department of Radiological Sciences, David Geffen School of Medicine, University of California-Los Angeles, United States of America; 2 Department of Biomedical Physics, David Geffen School of Medicine, University of California-Los Angeles, United States of America; 3 Department of Bioengineering, Henri Samueli School of Engineering and Applied Sciences, University of California-Los Angeles, United States of America; 4 Department of Psychiatry and Biobehavioral Sciences, David Geffen School of Medicine, University of California-Los Angeles, United States of America; 5 Department of Neurosurgery and Orthopaedics, David Geffen School of Medicine, University of California Los Angeles, Los Angeles, CA, United States of America; Toronto Western Hospital, CANADA

## Abstract

**Purpose:**

In the present study we investigated a combination of diffusion tensor imaging (DTI) and magnetic resonance spectroscopic (MRS) biomarkers in order to predict neurological impairment in patients with cervical spondylosis.

**Methods:**

Twenty-seven patients with cervical spondylosis were evaluated. DTI and single voxel MRS were performed in the cervical cord. N-acetylaspartate (NAA) and choline (Cho) metabolite concentration ratios with respect to creatine were quantified, as well as the ratio of choline to NAA. The modified mJOA scale was used as a measure of neurologic deficit. Linear regression was performed between DTI and MRS parameters and mJOA scores. Significant predictors from linear regression were used in a multiple linear regression model in order to improve prediction of mJOA. Parameters that did not add value to model performance were removed, then an optimized multiparametric model was established to predict mJOA.

**Results:**

Significant correlations were observed between the Torg-Pavlov ratio and FA (R^2^ = 0.2021, P = 0.019); DTI fiber tract density and FA, MD, Cho/NAA (R^2^ = 0.3412, P = 0.0014; R^2^ = 0.2112, P = 0.016; and R^2^ = 0.2352, P = 0.010 respectively); along with FA and Cho/NAA (R^2^ = 0.1695, P = 0.033). DTI fiber tract density, MD and FA at the site of compression, along with Cho/NAA at C2, were significantly correlated with mJOA score (R^2^ = 0.05939, P < 0.0001; R^2^ = 0.4739, P < 0.0001; R^2^ = 0.7034, P < 0.0001; R^2^ = 0.4649, P < 0.0001). A combination biomarker consisting of DTI fiber tract density, MD, and Cho/NAA showed the best prediction of mJOA (R^2^ = 0.8274, P<0.0001), with post-hoc tests suggesting fiber tract density, MD, and Cho/NAA were all significant contributors to predicting mJOA (P = 0.00053, P = 0.00085, and P = 0.0019, respectively).

**Conclusion:**

A linear combination of DTI and MRS measurements within the cervical spinal cord may be useful for accurately predicting neurological deficits in patients with cervical spondylosis. Additional studies may be necessary to validate these observations.

## Introduction

Cervical spondylosis is a common condition that occurs as a result of degeneration of the osseous and soft tissue structures within the spinal column.[1–4] Cervical spondylosis is present in a significant proportion of older individuals and myelopathy resulting from spondylosis is the most common cause of spinal cord dysfunction in the elderly [5]. As spondylosis is ubiquitous, the discernment between asymptomatic and potentially symptomatic individuals remains difficult, and a frequently debated area in our field. Decompression surgery is commonly performed in myelopathic patients, yet the response to surgery can significantly vary among individuals. As such, there is significant interest in the development of surrogate imaging biomarkers to non-invasively quantify neurological deficits, and predict which patients will obtain the best outcomes following surgical intervention.

Conventional magnetic resonance imaging (MRI) remains the best diagnostic tool for non-invasive assessment of the spinal cord owing to its sensitivity to soft tissue contrast; however, conventional MRI metrics thought to reflect spinal cord damage, including signal change [6–13] and the degree of spinal cord compression [14–16], have not demonstrated a strong association with neurological function. Diffusion tensor imaging (DTI), an advanced MR technique sensitive to the underlying microstructural organization of tissues [17], has previously shown to be valuable for predicting neurological function in patients with cervical spondylotic myelopathy (CSM).[18–24] In particular, studies have shown that lowered diffusion anisotropy at the site of compression is associated with increased neurological dysfunction, suggesting disruption in the directional coherence of nerve fibers in the spinal cord. Additionally, metabolic changes within the cervical spinal cord measured using MR spectroscopy (MRS) has been shown to be predictive of functional deficits in patients with CSM. Specifically, various studies have demonstrated an increase in choline and decrease in N-acetylaspartate in upper cervical spinal cord regions occur in spondylosis patients with neurological deficits. [25, 26] Based on these promising findings, we hypothesize that microstructural and metabolic data obtained using DTI and MRS, respectively, may provide distinct, yet complimentary information regarding the integrity of the spinal cord in patients with cervical spondylosis. Thus, the goal of the current study was to investigate a combination diffusion and metabolic MR biomarker for non-invasively predicting neurological function in patients with cervical stenosis with or without myelopathy.

## Materials and Methods

### Patient Population

A prospective study was performed in twenty-seven patients ranging from moderate to severe cervical stenosis, as demonstrated by narrowing of the spinal canal on standard MRI (Table 1; *n* = 27; 13 males and 14 males; age range 21–75; average age = 62). Moderate stenosis was defined as trace or no cerebrospinal fluid around the spinal cord without spinal cord deformity. Severe stenosis was defined as no cerebrospinal fluid around the spinal cord with spinal cord deformity. The modified Japanese Orthopaedic Association (mJOA)[27] was used as a qualitative measure of neurological impairment. Twenty-one patients had functional deficits (*n* = 21), as indicated by mJOA < 18, and six patients had no functional impairment (*n* = 6) but complaints of neck pain. The average mJOA score was 14.9 with a range from 8 to 18. All participants gave informed written consent to be part of this study. All procedures complied with the principles of the Declaration of Helsinki and were approved by the Institutional Review Board within the Office for the Protection of Research Subjects at the University of California (UCLA IRB#11-001876-AM-00004).

**Table 1:** Patient Clinical and Demographic Information. mJOA = Modified Japanese Orthopedic Association score. SEA = “Snake Eye” appearance.

	CLINICAL DATA				ANATOMIC MRI	
					T2	
Patient ID	Age	SEX	STENOSIS LEVEL	mJOA	Hyperintensity?	Snake Eyes?
1	71	F	C4-5 to C6-7	16	No	No
2	53	F	C4-5	17	No	No
3	53	M	C4-5 C5-6	13	No	No
4	54	F	C3-4 to C6-7	18	Yes	No
5	74	M	C3-4	17	No	No
6	21	F	C5-6 C6-7	17	No	No
7	69	M	C3-4 to C6-7	18	No	No
8	55	F	C3-4 to C5-6	15	No	No
9	58	M	C3-4 C4-5	8	Yes	Yes
10	66	M	C4-5	12	Yes	Yes
11	73	F	C4-5	18	No	No
12	62	M	C5-6	16	No	No
13	71	M	C4-5	14	No	No
14	75	M	C3-4 C4-5	16	No	No
15	69	F	C3-4 to C5-6	13	Yes	Yes
16	68	M	C4-5	12	No	No
17	39	F	C4-5	16	Yes	Yes
18	40	F	C3-4	12	Yes	Yes
19	57	F	C3-4	10	Yes	Yes
20	72	F	C3-4	13	Yes	No
21	72	M	C3-4 to C4-5	18	Yes	No
22	55	M	C3-4 to C5-6	18	No	No
23	58	F	C4-5	18	No	No
24	71	M	C3-4	10	Yes	Yes
25	72	F	C3-4 to C6-7	15	No	No
26	62	M	C3-4 and C5-6	15	No	No
27	74	F	C4 to C6	17	No	No

### Clinical Symptoms

Gait difficulty was the most common complaint at initial presentation and was observed in 11 of 27 patients. The duration of the gait deterioration ranged from three weeks to four years prior to their clinic evaluation. Hip pathology was ruled out in all patients that complained of gait difficulty. Ten patients suffered from difficulty using their hands consisting primarily of numbness and six patients noted paresthesias in their upper extremities. A history of neck pain was noted in 9 of 27 patients. Table 2 shows a summary of these symptoms.

**Table 2:** Presenting Symptoms.

Chief Complaint	Number of Patients
Gait Difficulty	11
Hand Incoordination	10
Neck Pain	9
Upper Extremity Parasthesis	6
Bladder Symptoms	2

### Conventional Magnetic Resonance Imaging

Standard MRI was obtained on a 3T MR scanner (3T Trio; Siemens Healthcare, Erlangen, Germany) using a standard spine coil array for radiofrequency reception. Routine clinical MRI scans consisted of T1-weighted and T2-weighted sequences in the sagittal plane and T2-weighted images in the axial orientation. All patients had radiographic evidence of stenosis, including spinal canal narrowing related to advanced cervical spondylosis manifested by a combination of facet arthropathy, ligamentum flavum hypertrophy, and varying degrees of ventral disc-osteophyte compression. The presence of T2 hyperintensity, presence of “snake eye appearance” (SEA) of T2 hyperintensity (bilateral T2 hyperintensity within the central cord),[28–31] anterior-posterior diameter of the cord at the level of highest compression, and the Torg-Pavlov ratio [14] (ratio of anterior-posterior diameter to thickness of the vertebral body) was documented and used for subsequent comparisons with diffusion and metabolic measurements.

### Diffusion Tensor Imaging (DTI)

Axial diffusion-weighted images were collected through the level of most significant canal narrowing. Excitation consisted of a custom two-dimensional, spatially selective radiofrequency excitation pulse (2D-RF) and a reduced FOV EPI readout with ramp sampling (Zoomed-EPI). The echo time (TE) / repetition time (TR) was set to 73ms/3000ms, matrix size = 48x128; field-of-view (FOV) = 53 mm x 140 mm, slice thickness = 4mm with no gap, number of averages = 10, and 20 diffusion sensitizing directions with *b* = 500 s/mm^2^ and 2-T2-weighted (*b* = 0 s/mm^2^) images. After acquisition of DWIs, eddy-current and motion correction was performed using a 12-degree of freedom affine transformation using FSL (FMRIB; Oxford, UK; http://www.fmrib.ox.ac.uk/fsl/). Mean diffusivity (MD), or average apparent diffusion coefficient relating to the mean water motility, as well as fractional anisotropy (FA), the degree of diffusion anisotropy, were calculated from the resulting diffusion MR data.

### DTI Fiber-Tracking and Tract Density Imaging (TDI)

Fiber-tracking was performed using the *MRtrix* software package (Brain Research Institute, Melbourne, Australia, http://www.brain.org.au/software/), based on the probabilistic streamlines method [32, 33] combined with the constrained spherical deconvolution (CSD) technique [34] to model multiple fiber-orientations. Tracking was performed by randomly seeding throughout the cervical spinal cord (1 million seeds placed throughout a 25 cm^3^ volume of the cervical cord, or 40,000 seeds per cm^3^) using a 0.1 mm step-size, a maximum angle between steps = 20 degrees, and a maximum harmonic order *I*_*max*_ *=* 4.

Quantitative maps of fiber tract density (tract density images, TDI) were created by first performing whole spine seeding (e.g. 1,000,000 seeds throughout the cervical cord), including areas of T2 hyperintensity that may be due to edema, inflammation, or myelomalacia. It is important to note that confounding factors that may cause myelopathy were excluded, including multiple sclerosis, transverse myelitis, and central cord syndrome. From the resulting fiber tracts, the total number of tracts present in each element on a high-resolution grid of 0.5mm isotropic resolution was calculated. These tract density images utilize the continuity information contained in the streamlines to quantify sub-voxel fiber tract density. The resulting quantitative tract density images were used for subsequent analysis.

### Magnetic Resonance Spectroscopy (MRS)

Single MR spectroscopy was obtained with a point resolved spectroscopy sequence with water suppression and pulse oximeter triggering to reduce artifacts due to movement of the spinal cord and cerebrospinal fluid pulsation. The following acquisition parameters were used: TR/TE = 2000ms/30ms, 256 averages, and a voxel size of 1.72mL (7mm x 7mm x 35mm). Water-enhanced T1 saturation effects were applied before the selected localization technique. Three frequency selective pulses were applied along with a spoiler gradient to suppress the water MR signal. A non-water suppressed MR spectrum was obtained with four averages for posthoc eddy current correction. The MRS voxel was placed at the posterior C2 vertebral level using sagittal T2-weighted images. We chose not to perform MRS at the site of compression based on our previous experience suggesting MRS at the site of compression results in inconsistent measurements due to: 1) magnetic susceptibility-related issues affecting local magnetic field homogeneity; 2) inconsistent volume of spinal tissue at the site of compression resulting in the need for substantially different voxel sizes across patients; and 3) increased motion artifact in lower cervical levels. Six saturation bands were placed around the MRS voxel for outer volume suppression. Manual shimming was performed to calculate optimal shim currents. Metabolite concentration ratios for choline to N-acetylaspartate (Cho/NAA), choline to total creatine (Cho/Cr), NAA/Cr, and myo-inositol to creatine (Myo-I/Cr) were calculated from the resulting peaks in the MR spectra.

### Regions of Interest

Manual segmentation of the spinal cord was performed for the whole cord (no gray/white matter distinction) at each axial image slice location using the T2-weighted anatomical images. DTI measurements at the site of highest compression, including voxels in areas of T2 hyperintensity, were used for comparison.

### Statistical Analysis

The linear relationship between anatomic, DTI, and MRS measurements and mJOA were determined using Pearson’s correlation coefficient. Next, the relationship between anatomic, DTI, and MRS measurements shown to be correlated with or thought to be associated with mJOA were determined using Pearson’s correlation coefficient. For these associations, statistical significance was determined by testing whether the slope of the linear trend line was significantly different from zero using an *F*-test. Lastly, multiple linear regression was performed using a model consisting of each imaging parameter shown to have a significant association with mJOA. *T*-tests were then performed on individual regression coefficients from the multiple linear regression model to determine which parameters added significant value to the prediction of mJOA. Parameters that were not statistically determined to have added value were then removed and the regression process was repeated, resulting in an optimal set of imaging parameters that could be used to predict mJOA score. All statistical analyses were performed using GraphPad Prism v6.0d (GraphPad Software, Inc., La Jolla, CA) and Matlab (R2011b; Mathworks, Inc., Natick, MA).

## Results

The mean spinal canal diameter was 6.7mm with a range of 3.9mm to 10.7mm. The mean Torg-Pavlo ratio was 0.4 with a range of 0.2 to 0.7. A total of 10 patients out of 27 had T2-weighted signal change within the spinal cord with 7 of these 10 patients exhibiting a characteristic “snake-eyes” appearance of signal change within the cord.

**Fig 1:**
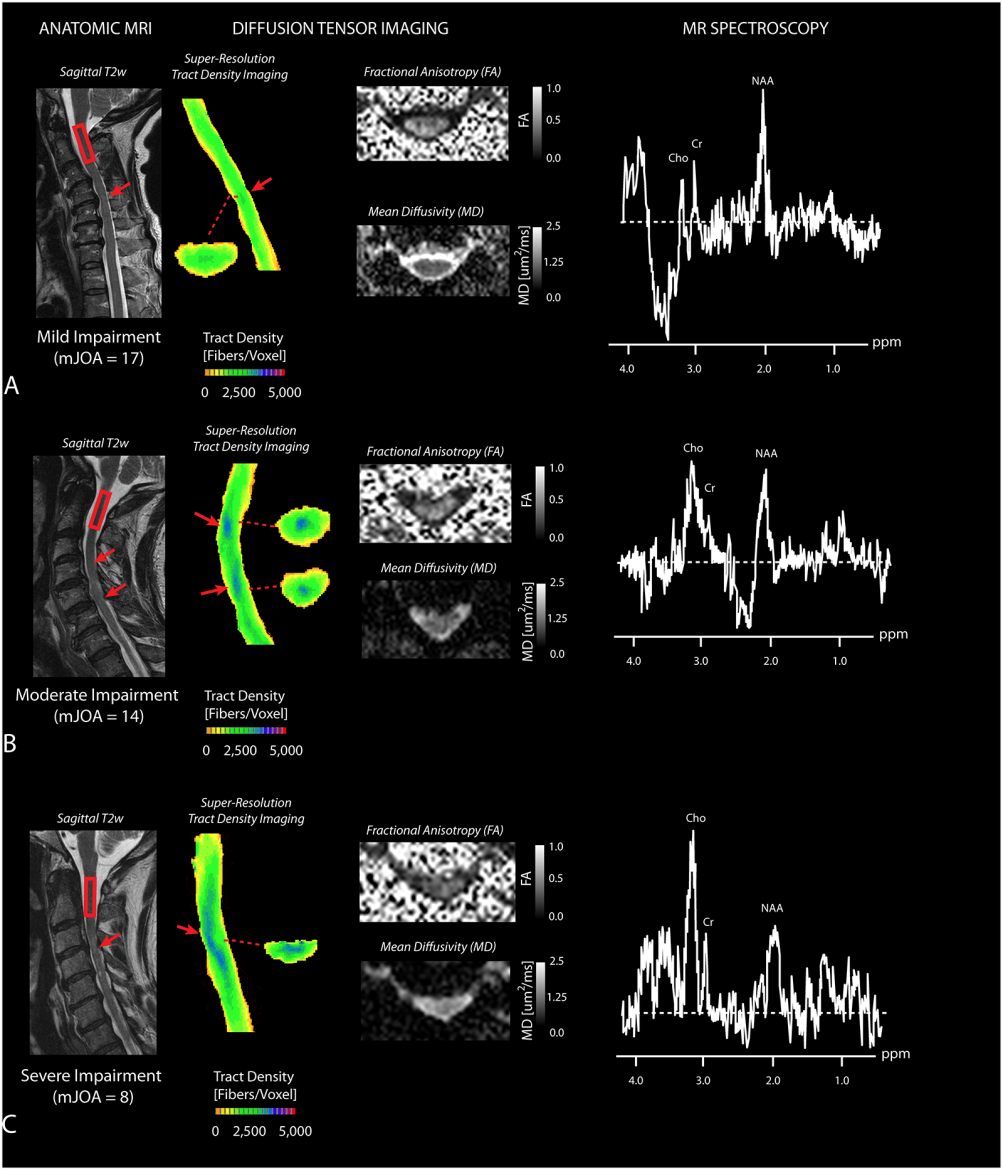
Anatomic, Diffusion, and Spectroscopic MR Measurements in Cervical Spondylosis. A) A patient with mild impairment (mJOA = 17) showing slightly elevated fiber tract density, relatively normal FA and MD, and a dominant *NAA* peak. B) A patient with moderate impairment (mJOA = 14) showing focally high fiber tract density, lower FA and higher MD, along with emergence of a *Cho* metabolites. C) A patient with severe impairment (mJOA = 8) showing elevated T2 hyperintensity on T2-weighted images, high fiber tract density indicative of compressed fibers extending rostral-caudal from the site of compression, lower FA and higher MD at the site of greatest stenosis, and dominant *Cho* peak in the MR spectra. Red box at C2 indicates the voxel used for MR spectroscopic measurement. Red arrow shows area of compression.

Patients with increasing neurological dysfunction appeared to have higher degrees of compression, T2 hyperintensity, lowered diffusion anisotropy, focally higher fiber tract density, and abnormal metabolic signatures compared with patients demonstrating stenosis without substantial neurological impairment (Fig 1; S1 Data). In particular, patients with a mild impairment (Fig 1A), defined as having mJOA scores between 15 and 17, tended to show only slightly increased fiber tract density in the area of compression, relatively high FA and low MD within the cord in the area of highest compression, and a dominant *NAA* peak with little *Cho* observed. In patients with moderate impairment (Fig 1B), or mJOA ranging from 12 to 14, focally higher fiber tract density was observed at the site of compression often extending rostral-caudal, lowered FA and slightly elevated MD was observed at the site of compression, and elevated *Cho* and lowered *NAA* levels were often noted. In patients with severe impairment (Fig 1C) exhibiting mJOA lower than 12, focally elevated fiber tract density was noted with significantly lower FA and higher MD present at the site of compression, along with a dominant *Cho* peak on MRS at the C2 level.

### Correlation Between Anatomic, Diffusion, and Spectroscopic MR Measurements

A significant linear correlation was observed between the Torg-Pavlov ratio and FA at the site of compression (Table 3; *R* ^*2*^ *= 0*.*2021*, *P = 0*.*019*); the ratio of maximum fiber tract density at the site of compression to fiber tract density at C2 and FA (*R* ^*2*^ *= 0*.*3412*, *P = 0*.*0014*), MD (*R* ^*2*^ *= 0*.*2112*, *P = 0*.*016*), and Cho/NAA (*R* ^*2*^ *= 0*.*2352*, *P = 0*.*010*); along with FA at the site of compression and Cho/NAA at C2 (*R* ^*2*^ *= 0*.*1695*, *P = 0*.*033*).

**Table 3:** Correlation (R^2^) Between Anatomic, Diffusion, and Spectroscopic MR Measurements.

	Age	Torg Ratio	Max TDI	FA	MD	Cho/NAA	Cho/Cr	NAA/Cr
**Age**	1.0000							
**Torg Ratio**	0.0087	1.0000						
**Max TDI**	0.0074	0.0878	1.0000					
**FA**	0.0029	**0.2021** *	**0.3412** **	1.0000				
**MD**	0.0001	0.0522	**0.2112** *	0.0851	1.0000			
**Cho/NAA**	0.0005	0.0068	**0.2352** *	**0.1695** *	0.1093	1.0000		
**Cho/Cr**	0.0058	0.0019	0.1138	0.0270	0.0796	0.0009	1.0000	
**NAA/Cr**	0.0200	0.0021	0.0487	0.0617	0.0039	0.0497	0.0016	1.0000

* = P < 0.05

** = P < 0.01

### Association Between mJOA and Anatomic, Diffusion, and Spectroscopic MR Measurements

**Fig 2:**
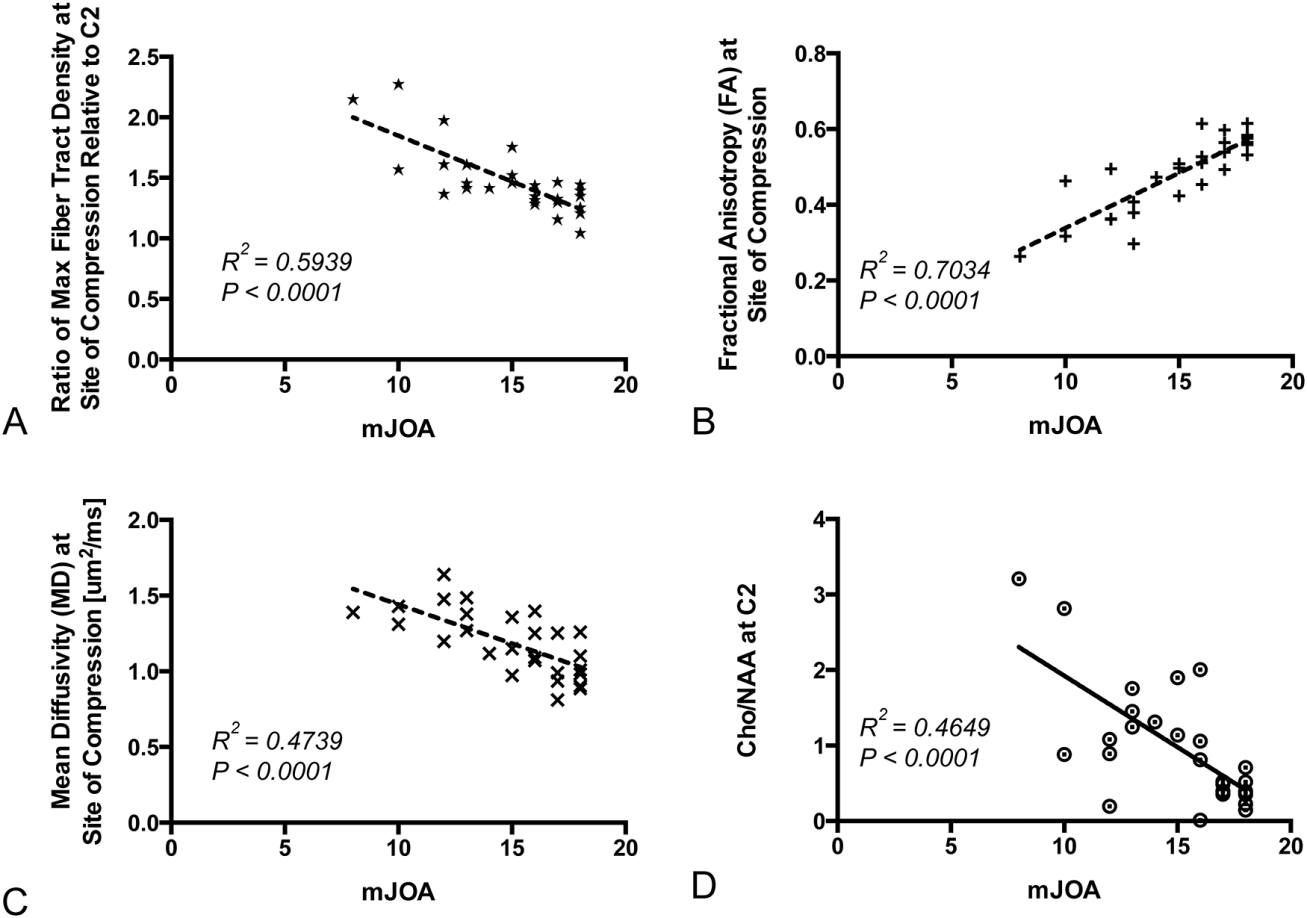
Correlation Between Individual MR Measurements and mJOA. A) Ratio of maximum fiber tract density at the site of compression to fiber tract density at C2 versus mJOA (*R* ^*2*^ *= 0*.*5939*, *P<0*.*0001*). B) Fractional anisotropy (FA) at the site of greatest stenosis versus mJOA (*R* ^*2*^ *= 0*.*7034*, *P<0*.*0001*). C) Mean diffusivity (MD) at the site of greatest stenosis versus mJOA (*R* ^*2*^ *= 0*.*4739*, *P<0*.*0001*). D) *Cho/NAA* ratio measured at C2 versus mJOA score (*R* ^*2*^ *= 0*.*4649*, *P<0*.*0001)*.

Results suggested no significant linear correlation between mJOA score and patient age (*R* ^*2*^ *<0*.*001*, *P = 0*.*9698*), vertebral body diameter at the site of compression (*R* ^*2*^ *= 0*.*093*, *P = 0*.*1223*), spinal cord diameter at the site of compression (*R* ^*2*^ *= 0*.*042*, *P = 0*.*3055*), Pavlov-Torg ratio (*R* ^*2*^ *= 0*.*1004*, *P = 0*.*1073*), Cho/Cr (*R* ^*2*^ *= 0*.*034*, *P = 0*.*3573*), NAA/Cr (*R* ^*2*^ *= 0*.*079*, *P = 0*.*1560*), or Myo-I/Cr (*R* ^*2*^ *= 0*.*019*, *P = 0*.*4965*). However, the ratio of maximum fiber tract density at the site of compression to fiber tract density at C2 (Fig 2A *R* ^*2*^ *= 0*.*5939*, *P<0*.*0001*), FA at the site of compression (Fig 2B; *R* ^*2*^ *= 0*.*7034*, *P<0*.*0001*), MD at the site of compression (Fig 2C; *R* ^*2*^ *= 0*.*4739*, *P<0*.*0001*), and Cho/NAA at C2 (Fig 2D; *R* ^*2*^ *= 0*.*4649*, *P<0*.*0001*) were all found to be correlated with mJOA after Bonferroni corrections for multiple comparisons.

### Combined Diffusion and Spectroscopic MR Biomarker for Predicting mJOA

**Fig 3:**
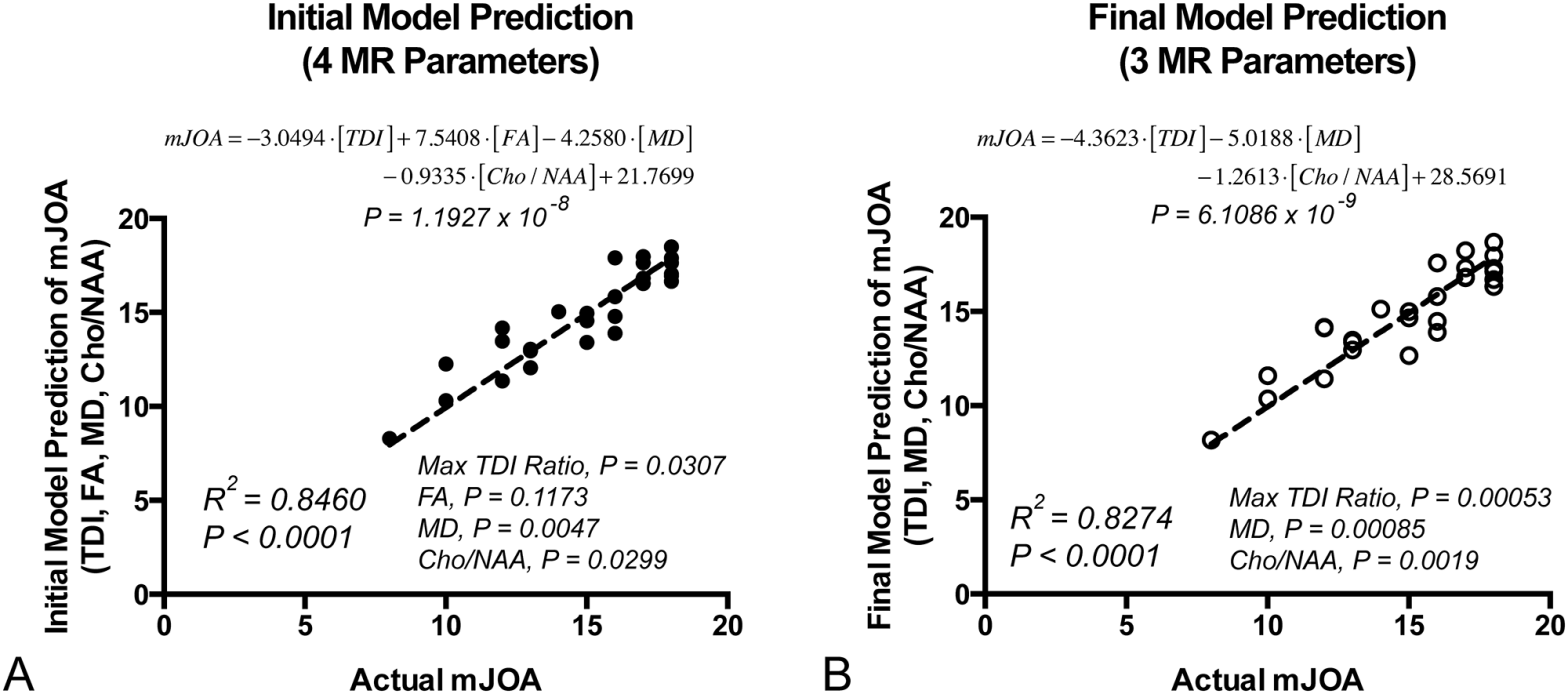
Initial and Final Combination Diffusion and Spectroscopic Biomarker Results for Prediction of mJOA. A) Initial model consisting of four MR measurements found to be individually correlated with mJOA. Note that results suggested *FA* at the site of compression did not add significant predictive value to the model. B) Final model consisting of three MR measurements (excluding FA).

Multiple linear regression was then performed using the individual imaging measurements determined to be significantly correlated with mJOA, namely the ratio of maximum tract density at the site of compression to tract density at C2, FA at the site of compression, MD at the site of compression, and Cho/NAA at C2. Results suggested a linear combination of these parameters was able to predict mJOA with high accuracy (Fig 3A; *P = 1*.*19x10* ^*-8*^) using the model *mJOA = -3*.*0494•[TDI] + 7*.*5408 •[FA]– 4*.*2580•[MD]– 0*.*9335•[Cho/NAA]+21*.*7699*, where *TDI* is the ratio of maximum fiber tract density at the site of compression to fiber tract density at C2, *FA* is the fractional anisotropy measured at the site of compression, *MD* is the mean diffusivity at the site of compression, and *Cho/NAA* is the measured Cho/NAA at C2. A high concordance was observed between the model predicted mJOA and actual mJOA (*R* ^*2*^ *= 0*.*8460*, *P<0*.*0001*). Individual *t*-tests on the regression coefficients showed that the ratio of maximum fiber tract density at the site of compression to fiber tract density at C2 (*P = 0*.*0307*), MD at the site of compression (*P = 0*.*0047*), and Cho/NAA at C2 (*P = 0*.*0299*) all added predictive value, whereas FA at the site of compression did not significantly contribute to added model performance (*P = 0*.*1173*). Since FA at the site of compression did not contribute significantly to the performance of the model, presumably due to the high correlation between FA and other parameters, this parameter was removed and the model was rerun. Results of this optimized model showed a higher accuracy in predicting mJOA (Fig 3B; *P = 6*.*11x10* ^*-9*^) using the model *mJOA = -4*.*3623•[TDI]– 5*.*0188•[MD]– 1*.*2613•[Cho/NAA]+28*.*5691*. A similarly high concordance was observed between the model predicted mJOA and actual mJOA (*R* ^*2*^ *= 0*.*8274*, *P<0*.*0001*) with this optimized model. Individual *t*-tests performed on the regression coefficients in the optimized model showed that the ratio of maximum fiber tract density (*P = 0*.*00053*), MD at the site of compression (*P = 0*.*00085*), and Cho/NAA (*P = 0*.*0019*) all provided added value for predicting mJOA score.

## Discussion

Spondylotic changes within the cervical spine are a normal part of aging, and are commonly encountered in clinical practice. One the major challenge in spine surgery is distinguishing between patients with advanced cervical spondylosis that is largely related to aging and generally benign, and those with advanced cervical spondylosis associated with current neurological impairment. Standard MRI provides macroanatomical information, which can appear quite similar radiographically between asymptomatic and symptomatic individuals including degree of spinal cord compression [14–16], spinal canal diameter, and even presence of spinal cord signal change. [6–13] One caveat to this ambiguity is the presence of SEA, or bilateral T2 hyperintensity within the central cord, which has previously been shown to correlate with current mJOA [35] and has been associated with worse prognosis after decompression surgery in patients with spinal cord compression.[28] As a result, there has been increasing interest in the development of novel imaging modalities that can better discern between asymptomatic individuals and those with varying degrees of disability.

Recent studies have suggested that advanced imaging techniques such as DTI [7, 18–20, 22, 24] and MRS [25, 26, 36, 37] may be invaluable for assaying microstructural and biochemical information related to degeneration and metabolic dysfunction, and provide more sensitive identification of spinal cord injury than standard MRI. DTI and MRS offer distinct, yet complementary information regarding microcellular injury that can be detected in the absence of clear macroscopic damage to the spinal cord. DTI evaluates diffusion anisotropy, in which diffusion occurs in a preferred direction in central nervous system white matter, and is largely attributed to the transverse axonal barriers in white matter (i.e. myelin sheath, axon membrane, etc.), restricting water diffusion to a preferred longitudinal orientation. Reduced diffusion anisotropy at the site of injury indicates disturbance in the directional orientation of nerve fibers in the spinal cord, and is associated with increased neurological impairment. In contrast, MRS analyzes the relative concentrations of critical biochemical metabolites, which can provide insight into cellular function and homeostasis. Although a variety of metabolites can be measured, NAA and choline are of particular importance. A decrease in NAA is a well known biomarker for neuronal and axonal injury, and an increase in choline secondary to cellular membrane injury have both been described in various CNS pathological conditions [38–41]. The finding of an increased Cho/NAA correlating with functional decline is consistent with our previous findings [25, 26, 37], and may imply a subtle downstream, propagating degenerative process (i.e. Wallerian degeneration) as reflected by a combination of axonal loss, metabolic dysfunction (decreased NAA) or increased membrane turnover (increased Cho).

In the present study we sought to investigate the utility of a combination of DTI and MRS to evaluate spinal cord injury in a cohort of patients with advanced cervical spondylosis. The use of concurrent DTI and MRS to evaluate pathological conditions of the CNS has been previously described for neoplastic disorders [42]and upper motor neuron disease [43]. However, these previous investigations involved cranial imaging, and not the spine. Independently, we found that both advanced imaging modalities correlated with degree of neurological impairment. DTI demonstrated that the ratio of maximum fiber tract density at the site of compression to fiber tract density at C2 (TDI), FA at the site of compression, and MD at the site of compression were all significantly correlated with mJOA score. A higher fiber tract density, lower FA, and higher MD were associated with poorer neurological function. As for MRS, the Cho/NAA ratio was significantly associated with mJOA. This significant correlation is likely due to the fact that the Cho/NAA ratio probes both the integrity of axons and neurons as well as the degree of cellular injury and turnover.

The combination DTI/MRS biomarker model provided an even stronger correlation to mJOA score and degree of neurological impairment than either modality alone. The model included the DTI parameters TDI, FA, and MD, as well as the MRS biomarker Cho/NAA ratio. The simultaneous assessment of microstructural and metabolic injury provided pertinent information that synergistically functioned to sharpen the predictive value of either independent modality. Although FA was found to be significantly associated with mJOA in the current study as well as previous studies, inclusion of FA in the composite model was not found to add significant value to the prediction of mJOA. Presumably, this lack of added value was due to the strong correlation between FA and other important parameters, including maximum track density and Cho/NAA. Thus, after removing FA, the DTI/MRS combination model was an even better predictor of neurological impairment, as suggested by the higher level of statistical significance (i.e. lower *p*-value).

The aforementioned studies evaluated both cranial MRS and DTI and demonstrated agreement between individual modalities and neurological status. However, the relevant MRS and DTI measurements were evaluated in parallel, and not truly combined. In addition to the novelty associated with applying both cervical spine MRS and DTI concurrently in individual CSM patients, the present study demonstrated potential value of a direct combination of both modalities into a single metric which would be used to assay neurological impairment in CSM patients. Future studies in a larger cohort of patients will be needed to prospectively validate this model.

### Limitations

Although DTI and MRS techniques provide novel insights into the health of the spinal cord there remains significant limitations to widespread use. These techniques are technically challenging to employ largely related to the small size and movement of the cord. One limitation of the current study is the acquisition of reliable MR spectroscopic data within the spinal cord, as motion or cord movement, difficulty in magnetic field shimming, and partial volume contamination can cause inaccuracies in MRS measurements. To overcome these challenges we chose to perform MRS at the C2 level instead of the site of compression. The placement of this voxel, however, may have resulted in metabolic concentrations/ratios that are dependent on both the severity of myelopathy as well as the distance from the site of compression. Another potential limitation to the present study was the use of only 20 diffusion-sensitizing directions to estimate the diffusion tensor and fiber track density images. Although previous spinal cord DTI studies have suggested that white matter integrity assessment in the spinal cord may not require a full tensor [44], DTI studies in the brain have shown increased accuracy of the tensor with increasing diffusion directions [45, 46]. Future studies aimed at characterizing these potential limitations are warranted in order to better understand their implications on predicting neurological dysfunction.

## Supporting Information

S1 Data Clinical data, conventional MRI measurements, diffusion tensor MRI measurements, and MR spectroscopic metabolite ratios for all 27 patients with cervical spondylosis included in the current study. (XLS)
